# TNF-alpha promotes lymphangiogenesis and lymphatic metastasis of gallbladder cancer through the ERK1/2/AP-1/VEGF-D pathway

**DOI:** 10.1186/s12885-016-2259-4

**Published:** 2016-03-19

**Authors:** HaiJie Hong, Lei Jiang, YanFei Lin, CaiLong He, GuangWei Zhu, Qiang Du, XiaoQian Wang, FeiFei She, YanLing Chen

**Affiliations:** Department of Hepatobiliary Surgery and Fujian Institute of Hepatobiliary Surgery, Fujian Medical University Union Hospital, 29 Xinquan Road, Fuzhou, 350001 China; Key Laboratory of Ministry of Education for Gastrointestinal Cancer, Fujian Medical University, 1 Xueyuan Road, Minhou, Fuzhou, 350108 China; Fujian Key Laboratory of Tumor Microbiology, Fujian Medical University, 1 Xueyuan Road, Minhou, Fuzhou, 350108 China

**Keywords:** Gallbladder cancer, TNF-α, VEGF-D, Lymphatic metastasis

## Abstract

**Background:**

Tumor necrosis factor-alpha (TNF-α), a key player in cancer-related inflammation, was recently demonstrated to be involved in the lymphatic metastasis of gallbladder cancer (GBC). Vascular endothelial growth factor D (VEGF-D) is a key lymphangiogenic factor that is associated with lymphangiogenesis and lymph node metastasis in GBC. However, whether VEGF-D is involved in TNF-α-induced lymphatic metastasis of GBC remains undetermined.

**Methods:**

The expression of VEGF-D in patient specimens was detected by immunohistochemistry and the relationship between VEGF-D in the tissue and TNF-α in the bile of the matching patients was analyzed. The VEGF-D mRNA and protein levels after treatment with exogenous TNF-α in NOZ, GBC-SD and SGC-996 cell lines were measured by real-time PCR and ELISA. The promoter activity and transcriptional regulation of VEGF-D were analyzed with the relative luciferase reporter assay, mutant constructs, electrophoretic mobility shift assay (EMSA), chromatin immunoprecipitation (ChIP) assay, RNA interference and Western blotting. Inhibitors of JNK, p38 MAPK and ERK1/2 were used to explore the upstream signaling effector of AP-1. We used lentiviral vector expressing a VEGF-D shRNA construct to knockdown VEGF-D gene in NOZ and GBC-SD cells. The role of the TNF-α-VEGF-D axis in the tube formation of human dermal lymphatic endothelial cells (HDLECs) was determined using a three-dimensional coculture system. The role of the TNF-α - VEGF-D axis in lymphangiogenesis and lymph node metastasis was studied via animal experiment.

**Results:**

TNF-α levels in the bile of GBC patients were positively correlated with VEGF-D expression in the clinical specimens. TNF-α can upregulate the protein expression and promoter activity of VEGF-D through the ERK1/2 - AP-1 pathway. Moreover, TNF-α can promote tube formation of HDLECs, lymphangiogenesis and lymph node metastasis of GBC by upregulation of VEGF-D in vitro and in vivo.

**Conclusion:**

Taken together, our data suggest that TNF-α can promote lymphangiogenesis and lymphatic metastasis of GBC through the ERK1/2/AP-1/VEGF-D pathway.

## Background

Gallbladder cancer (GBC) is rare but represents the most common cancer of the biliary tract, accounting for 80–95 % of biliary tract malignancies [1, 2]. GBC is a highly aggressive disease with very poor prognosis (5-year survival rate < 5 % [3, 4]), due to its tendency to metastasize to the lymph nodes in early stages. More than 50 % of all patients with GBC exhibit lymph node metastases (LNM) [5]. Therefore, understanding the mechanism underlying lymphatic metastasis in GBC is helpful to improve patient treatment and prognosis. However, the specific mechanisms underlying lymphatic metastasis in GBC are largely unknown.

In 1863, Virchow first observed that inflammatory cells can be found in tumors [6]. Since then, many studies have examined the relationship between inflammation and cancer. It has been generally accepted that chronic inflammation promotes cancer [7], including some cancers of the liver [8], intestine [9, 10] and lung [11]. Cytokines secreted by inflammatory cells, including TNF-α, IL-1, and IL-6, play important roles in cancer-related inflammation [7, 12–15]. Tumor necrosis factor alpha (TNF-α), a key pro-inflammatory cytokine that was first identified as a mediator of tumor cell death, is now also known to promote the tumor progression, proliferation, epidermal-mesenchymal transition (EMT), angiogenesis, invasion and metastasis [16–19]. Lymphatic metastasis is one of the major forms of tumor metastasis. However, the relationship between TNF-α and lymphatic metastasis requires further research.

Recently, we confirmed that TNF-α can promote lymphangiogenesis and lymph node metastasis of GBC through upregulation of vascular endothelial growth factor C (VEGF-C) downstream of NF-κB [20]. Furthermore, we determined that vascular endothelial growth factor D (VEGF-D), another key lymphangiogenic factor similar to VEGF-C, is associated with lymphangiogenesis and lymph node metastasis of GBC [21]. Thus, we aimed to further explore whether VEGF-D is involved in TNF-α-induced lymphatic metastasis of GBC and the underlying mechanisms.

In this study, we first analyzed the relationship between TNF-α levels and VEGF-D expression in clinical specimens and demonstrated that TNF-α can upregulate VEGF-D expression in the NOZ and GBC-SD cell lines. Previous studies have demonstrated that TNF-α promotes the expression of target genes mainly through NF-κB and (or) AP-1 signaling pathways [22]. We further sought to determine whether TNF-α upregulates VEGF-D expression and enhances its promoter activity through these two pathways. Furthermore, we determined that TNF-α can promote tube formation of human dermal lymphatic endothelial cells (HDLECs), lymphangiogenesis and lymph node metastasis of GBC by upregulation of VEGF-D *in vitro* and *in vivo*.

## Methods

### Patient samples and cell culture

20 GBC tissues and the matching bile used in present study were obtained from the patients admitted to Fujian Medical University Union Hospital in China. The informed consents of agreement to use the samples for further study were signed pre-operation. The samples were collected according to the protocol approved by the Ethics Committee of the Medical Faculty of Fujian Medical University, according to the Declaration of Helsinki. The details of the patients including the age and sex of the patient, clinical stage, grade of the tumor and lymph node metastasis (LNM) had been described in [20]. The human GBC cell lines: NOZ (obtained from Health Science Research Resources Bank in Japan), GBC-SD (purchased from Shanghai Institutes for BiologicalSciences in China) and SGC-996 (provided by the Tumor Cytology Research Unit, Medical College, Tongji University, China) were maintained in Dulbecco’s Modified Eagle’s Medium (Gibco, USA) supplemented with 10 % fetal bovine serum (Gibco). Human dermal lymphatic endothelial cells (HDLECs, purchased from Sciencell, San Diego, California, USA) were incubated in endothelial cell medium (Sciencell). All of the cells were incubated at 37 °C under 95 % air and 5 % CO2.

### Immunohistochemistry

The VEGF-D expression and lymphatic vessels of GBC specimens were detected by immunohistochemistry as previously described [21]. The primary antibodies were VEGF-D (ab155288, Abcam) at a 1:80 dilution and LYVE-1 (AF2125, R&D Systems) at a 1:150 dilution. The method used to measure the VEGF-D expression has been described previously [23]. The density of LYVE-1-positive vessels (lymphatic vessels density, LVD) was assessed according to the method described by Qiang Du [24].

### Quantitative real-time polymerase chain reaction (qRT-PCR)

Total RNA was extracted from GBC cells with TRIzol reagent (Invitrogen). RNA was reverse transcribed using the RevertAid First Strand cDNA Synthesis Kit (Thermo). PCR reactions were performed with Fast Start Universal SYBR Green Master Mix (Roche), and fluorescence was measured using the 7500 quantitative real-time thermocycler (Applied Biosystems). GAPDH served as an internal control. All procedures followed the manufacturer’s instructions.

### Enzyme-linked immunosorbent assay (ELISA)

VEGF-D levels in cell culture media were measured by double antibody sandwich enzyme-linked immunosorbent assay using Quantikine ELISA Kits from R&D Systems following the manufacturer’s instructions. VEGF-D Standards for drawing standard curve were prepared before the antibody reaction. 100 μL of Assay Diluent RD1X was added to each well, and then 50 μL of Standard, sample or control were added to each well and incubated for 2 h at room temperature. Wash each well with wash buffer (400 μL) for four times. Add 200 μL of VEGF-D Conjugate to each well and incubate for 2 h at room temperature. Wash each well again and add 200 μL of Substrate Solution to each well. Add 50 μL of Stop Solution to each well after incubation for 30 min (protect from light). The wells were read at 450 nm with a Model 550 Microplate Reader (Bio-Rad, Hercules, CA, USA). Each reaction was run in triplicate.

### Construction of VEGF-D promoter luciferase reporter plasmids and dual-luciferase reporter assay

A series of 5′-deletion DNA fragments of the VEGF-D gene promoter were amplified by PCR with primers containing an XhoI or BglII (Thermo) restriction site, which were connected to the pGL4.10-Basic vector (Promega) carrying a firefly luciferase report gene. These recombinant VEGF-D promoter luciferase reporter plasmids were named PGL4-2148 (−2148 to +117, relative to the transcription start site “ATG”), PGL4-1621 (−1621 to +117), PGL4-988 (−988 to +117), PGL4-717 (−717 to +117), PGL4-444 (−444 to +117), PGL4-325 (−325 to +117), PGL4-154 (−154 to +117), and PGL4-57 (−57 to +117). Forty-eight hours after transfection with promoter vector, cells were lysed and the intracellular luciferase activity of the lysates was measured using the Dual-Luciferase Reporter Assay System (Promega) according to the manufacturer’s instructions. The relative luciferase units were obtained by comparison with the luciferase activity of the pRL-TK plasmid (plasmid carrying a renilla luciferase report gene as an internal reference).

### Identification of putative transcription factor binding sites

The websites TFbind (http://tfbind.hgc.jp/) and Promoter Scan (http://www-bimas.cit.nih.gov/molbio/proscan/) were used to search for potential transcription factor binding site motifs.

### Site-directed mutagenesis

The site-directed mutagenesis was performed by overlap extension PCR as previously described [20, 25]. The primers targeting the two mutation sites of the AP-1 binding sites were as follows: AP-1mut1 (−401 to -393 nt), (forward), 5′-CATCTGCTGCCAATGCTACACAGAAAGCAATC-3′ (reverse); AP-1mut2 (−345 to -337 nt), 5′-CTTAAGCAATCCCACCGAGATACAAAGGTC-3′ (forward), 5′-GACCTTTGTATCTCGGTGGGATTGCTTAAG-3′ (reverse).

### Nuclear extraction and electrophoretic mobility shift assay (EMSA)

Nuclear proteins were extracted from NOZ cells using the Nuclear and Cytoplasmic Protein Extraction Kit (Beyotime, JiangSu, China), and electrophoretic mobility shift assay (EMSA) was performed with the LightShift Chemiluminescent EMSA kit (Thermo Scientific, Inc.) according to the manufacturers’ recommendations. Two biotin-labeled oligonucleotide probes (5′biotin-CTTTCTGTGTGTCATTGGCAG-3′, which contained −401 to −393 nt, and 5′biotin-ATCCCACTGAGATACAAAGGT-3′, which contained −345 to −337 nt) were used to confirm the DNA binding of AP-1. For competition analysis, we used 100-fold excess of unlabeled competitive probes, including cold probes and mutational cold probes (5′-CTTTCTGTGTAGCATTGGCAG-3′, and 5′-ATCCCACCGAGATACAAAGGT-3′, mutation sites underlined).

### Chromatin immunoprecipitation (ChIP) assay

The ChIP assay was performed according to the manufacturer’s instructions using the EZ-Magna ChIP kit (Merck Millipore, Darmstadt, Germany). An antibody against AP-1 (c-Jun, phosphor S63, Abcam), a negative control normal rabbit IgG, and a positive control anti-acetyl histone H3 antibody were used for immunoprecipitation. The primers for PCR were as follows: 5′-TTGCATGTATGGATGGATGTTTT-3′ (forward) and 5′-AAGAAGGGACCTCAGATGCTCAT-3′ (reverse); and 5′-GAGCATCTGAGGTCCCTTCTTAA-3′ (forward) and 5′-AAGAAGGGACCTCAGATGCTCAT-3′ (reverse).

### AP-1(c-Jun) siRNA oligonucleotide treatment of cells

The AP-1 (c-Jun) siRNA interference sequence has been described previously [26] (named siAP-1, sense: 5′-GAUGGAAACGACCUUCUAUdTdT-3′, anti-sense: 5′-AUAGAAGGUCGUUUCCAUCdTdT-3′), and the non-targeting control (named siNC) were synthesized chemically by GenePharma Co., Ltd. (Suzhou, China). The transient transfection was performed according to the manufacturer’s instructions.

### Western blotting

Western blot analysis was performed as described previously [27]. Cells were washed twice with ice cold PBS and then incubated on ice with 100 μL of RIPA buffer with 100 mM PMSF (phenylmethylsulfonyl fluoride) for 15 min. Plates were scraped and lysates were centrifuged at 13,000 rpm for 5 min at 4 °C. The protein concentrations of cell lysates were measured in duplicate using a BCA Protein Assay Kit (Beyotime Institute of Biotechnology, Shanghai, China). The appropriate amount of 5× loading buffer was mixed with the protein lysates and boiled for 5 min at 100 °C. Equal amounts of total protein were resolved by 10 % SDS (sodium dodecyl sulfate)-polyacrylamide gel electrophoresis and transferred to PVDF (polyvinylidene fluoride) membranes. The PVDF membranes were then blocked with 5 % nonfat milk in Tris Buffered Saline with Tween (TBST; 10 mM Tris–HCl, 150 mM NaCl, and 0.05 % Tween) for 2.5 h. The appropriate diluted primary antibodies, including anti-VEGF-D, anti-AP-1 (c-Jun, phospho-S63), anti-phosphorylated AP-1 (p-AP-1) antibodies (1:1000, Abcam) and the β-actin antibody (1:1000, Santa Cruz), were then incubated with the membranes overnight at 4 °C. The appropriate secondary antibody conjugated with horseradish peroxidase diluted in TBST was added for 1 h at room temperature. Immunoreactivity was detected using a chemiluminescence western blot immunodetection kit (Invitrogen) according to the manufacturer’s instructions and recorded on Hyperfine-ECL detection film. The amounts of each protein were semiquantified as ratios to β-actin indicated on each gel.

### Construction of a stable NOZ cell line with lentiviral VEGF-D shRNA

We previously identified an siRNA sequence (5′-GCUAUGGGAUAGCAACAAAUG-3′) that effectively knocked down VEGF-D gene expression in NOZ cells [21]. To establish a stably expressing cell line, we used lentiviral vector expressing a VEGF-D shRNA construct (named LV-siVEGF-D) and a control vector containing a non-targeting sequence (named LV-siNC). Both vectors were constructed by Genepharma Co., Ltd. (Suzhou, China) and were used to infect NOZ and GBC-SD cells; puromycin was used to screen for stably infected cells.

### Tube formation assay

To assess the role of the TNF-α-VEGF-D axis in the tube formation of HDLECs, NOZ or GBC-SD cells stably transfected with LV-siVEGF-D were co-cultured with HDLECs previously labeled by DiI (a cell membrane dye emitting red fluorescence; Beyotime Institute of Biotechnology, ShangHai, China) in a three dimensional coculture system following the method described by Yiping Zeng [28]. Briefly, 7.5 × 10^3^/well of GBC cells and 7.5 × 10^3^/well of HDLECs were seeded to the same well of microwell-plate (ibidi) which was previously painted with matrigel. Tube formation of HDLECs was observed by inverted fluorescence microscopy (Nikon, Japan), and images were digitally captured at 1 h, 3 h, 5 h, 8 h and 24 h after cell seeding. The total number of tube-like structures formed in each well were measured with Axiovision Rel 4.1 software (Carl Zeiss AG, Jena, Germany).

### Establishment of the orthotopic xenograft model

Thirty male athymic BALB⁄c nude mice 4–6 weeks-old were obtained from Slaccas Laboratory Animal Co. (Shanghai, China) and raised in the specefic pathogen free (SPF) laboratory animal room. All experiments in this part were carried out in accordance with institutional guidelines and were approved by the Ethics Committee of the Medical Faculty of the Fujian Medical University. The orthotopic xenograft models were established following the method by Qiang Du [20, 24]. Two weeks later, exogenous TNF-α (2 μg/kg) was injected into the peritoneal cavity every 3 days for 3 weeks. Five weeks after injection of cells, the mice were euthanized by exposure to CO_2_, and primary tumors were dissected and excised.

### Statistics

Results are presented as the mean ± SD from at least three independent experiments. Data were analyzed by Student’s t-test. A two-sided *P*-value <0.05 was considered statistically significant.

## Results

### VEGF-D expression in human GBC and the relationship between VEGF-D and TNF-α

**Fig. 1:**
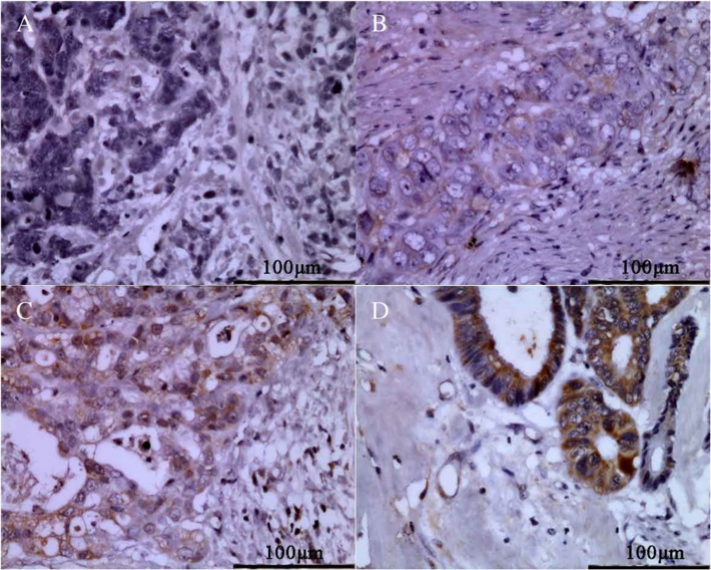
Representative IHC stainging examples demonstrating VEGF-D expression in GBC specimens: **a** absent, **b** weak, **c** moderate, **d** strong

Our previous study demonstrated that the level of TNF-α in the bile of GBC patients was significantly higher than that in patients with cholesterol gallbladder polyps [20]. To examine the expression of VEGF-D in human GBC samples and analyze the relationship between VEGF-D and TNF-α, we used immunohistochemistry to detect the expression of VEGF-D in 20 GBC samples. The TNF-α levels in the bile of these patients had been detected by ELISA in our previous study [20]. As shown in Fig. 1, The VEGF-D protein was stained as light to dark brown and is mainly located in the cytoplasm of GBC cells. As shown in Table 1, VEGF-D was expressed in 75 % (15/20) of samples. The level of TNF-α in the bile of GBC patients with positive staining of VEGF-D was significantly higher than that of patients with negative staining.

**Table 1:** The relationship between TNF-α levels in the bile and VEGF-D expression in the tissues of GBC patients

VEGF-D	Case number	TNF-α (pg/ml)	*P* value
Positive	15	666.71 ± 47.26	0.017
Negative	5	435.98 ± 49.08	

### TNF-α promotes the expression of VEGF-D *in vitro*

**Fig. 2:**
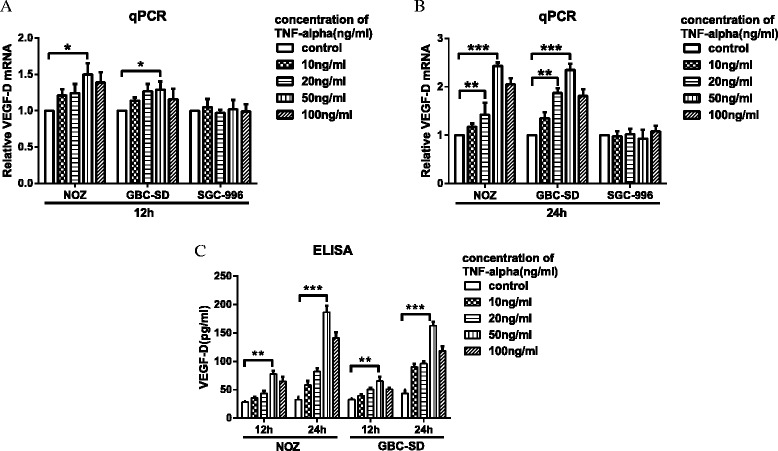
VEGF-D mRNA transcription and protein expression in three GBC cell lines after treatment with TNF-α. GBC cells (NOZ, GBC-SD, and SGC-996) were treated with varying concentrations of TNF-α (10, 20, 50 and 100 ng⁄ mL) for 12 or 24 h. The VEGF-D mRNA and protein levels were measured by real-time PCR (**a**, **b**) and ELISA (**c**), respectively, and increased in a dose- and time-dependent manner in NOZ and GBC-SD cell lines but not SGC-996 cells. (*P < 0.05; **P < 0.01; ***P < 0.001)

To determine whether TNF-α could promote the expression of VEGF-D, we measured the expression of VEGF-D in three GBC cell lines (NOZ, GBC-SD, and SGC-996) after treatment with exogenous TNF-α. GBC cells were incubated in 6-well plates and treated with varying doses of TNF-α (10, 20, 50 and 100 ng⁄mL) for 12 and 24 h; the control samples were not treated with TNF-α. The relative mRNA of VEGF-D was assayed by real-time PCR, and VEGF-D protein level in the cell culture supernate was detected by ELISA. As shown in Fig. 2, TNF-α promoted the transcription and protein expression of VEGF-D in NOZ and GBC-SD cell lines (but not SGC-996 cells) in a dose- and time-dependent manner, and the peak effect appeared after 24-h treatment with 50 ng⁄mL TNF-α. So we used NOZ and GBC-SD cells to next further study.

### Activity analysis of VEGF-D promoter

**Fig. 3:**
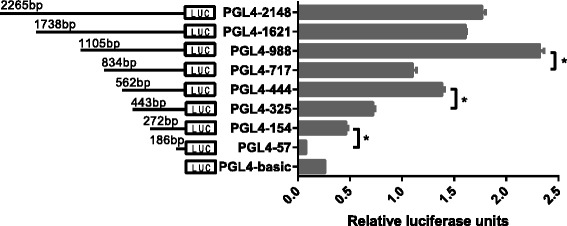
Activity analysis of VEGF-D promoter. A series of 5′-deletion fragments of the VEGF-D promoter were amplified by PCR and then inserted into the firefly luciferase report vector. These constructs (1 μg) were co-transfected into NOZ cells with pRL-TK (0.1 μg) as an internal reference. PGL4-basic served as the negative control. The constructs PGL4-988, PGL4-444, and PGL4-154 exhibited higher relative luciferase activities (compared with PGL4-717, PGL4-325, and PGL4-57, respectively (*P < 0.05)). The experiment was repeated three times

To further explore the mechanism by which TNF-α upregulates VEGF-D, we analyzed the promoter of VEGF-D. Recombinant plasmids carrying a series of 5′-deletion fragments of the VEGF-D gene promoter and the firefly luciferase report gene (named PGL4-2148, PGL4-1621, PGL4-988, PGL4-717, PGL4-444, PGL4-325, PGL4-154, and PGL4-57) were transiently co-transfected into the NOZ cells with pRL-TK as internal reference. As shown in Fig. 3, cells transfected with recombinant plasmids PGL4-988, PGL4-444, and PGL4-154 exhibited higher relative luciferase activities compared with cells transfected with PGL4-717, PGL4-325, and PGL4-57, respectively (*P* < 0.05). Therefore, we speculated that the three fragments (−988 to −71 7 nt,-444 to -325 nt,and −154 to -57 nt) contained sites regulating VEGF-D expression. Next, we scanned the base sequences of the fragments using the TFbind and Promoter Scan programs to search for potential binding sites of the transcription factor AP-1 and NF-κB. The region −444 to -325 nt contained two putative AP-1 binding sites but no NF-κB site, and neither of the other two regions contained binding sites. The plasmid PGL4-444 was therefore selected for further studies.

### TNF-α promotes AP-1 binding to the VEGF-D promoter

**Fig. 4:**
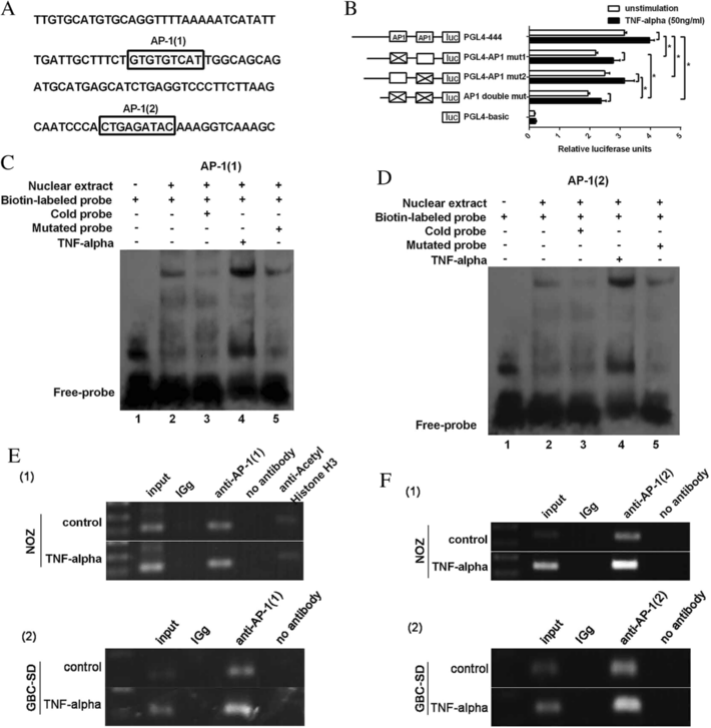
TNF-α promotes AP-1 binding to the VEGF-D promoter. **a**. The two predicted putative AP1-binding sites contained in the −444 to −325 nt region of VEGF-D promoter are underlined (AP-1(1) in the nucleotide region −401 to -393 nt; AP-1(2) in the −345 to -337 nt). **b**. The effect of mutation of the AP-1 binding sites on the activity of VEGF-D promoter. Both of the two mutated constructs, PGL4-AP-1mut1 and PGL4-AP-1mut2, exhibited lower activities than the non-mutated construct PGL4-444. Furthermore, the activity weakened when the two sites were mutated simultaneously. The trend persisted upon treatment with TNF-α (50 ng/ml) (mutants, indicated with the × mark, are depicted schematically on the left; *P < 0.05). **c**, **d**. EMSA of AP-1. The nuclear extracts from NOZ cells could bind the biotin-labeled probes (lane 2). The competition assay revealed that pre-incubation with the cold probes (lane 3) but not the cold mutated probes (lane 5) diminished the intensity of the bands. TNF-α enhanced the combined effect of the nuclear extracts and the two AP-1-binding sites (lane 4). **e**, **f**. ChIP assay. Chromatin from NOZ or GBC-SD cells was immunoprecipitated with the anti-AP-1 antibody. The total extracted DNA (Input) and the immunoprecipitated samples were PCR-amplified using primers specific to the regions of the VEGF-D promoter containing the AP-1(1) binding site (119 bp) and AP-1(2) binding site (150 bp). A normal rabbit IgG and no antibody sample were also included as controls. Another experiment group was treated with 50 ng⁄ mL of TNF-α (bottom row), and TNF-α enhanced the intensity of the input and anti-AP-1 bands

The sequence of the −444 to −325 nt region of the VEGF-D promoter is presented in Fig. 4a, and the two predicted putative AP1-binding sites in the nucleotide region −401 to −393 (AP-1(1)) and −345 to −337 (AP-1(2)) are underlined. Site-directed mutants of the putative AP1-binding sites were then generated, and the promoter activities of the corresponding constructs were measured. As shown in Fig. 4b, both of the two recombinant plasmids, PGL4-AP-1mut1, which contains the mutation of the AP-1(1)-binding site, and PGL4-AP-1mut2, which contains the mutation of the AP-1(2)-binding site, exhibited lower activities than the control non-mutated construct (PGL4-444). Furthermore, the activity weakened when the two sites were mutated simultaneously (AP-1 double mut), which suggests that both of the AP-1-binding sites are crucial for the full activity of the VEGF-D promoter. Upon treatment with TNF-α, the activity of PGL4-444 increased significantly (*P* < 0.05), and this activity was impaired by the mutation of the AP-1-binding sites.

The two AP-1 binding sites were further confirmed by EMSA of nuclear extracts from NOZ cells with and without TNF-α treatment. As shown in Fig. 4c, the nuclear extracts were combined with a biotin-labeled probe (lane2). A competition assay revealed that pre-incubation with a 100-fold molar excess of the cold probe (lane3) but not the cold mutated probe (lane5) diminished the intensity of the bands. Moreover, TNF-α enhanced the combined effect of the nuclear extract and the AP-1(1)-binding site (lane 4). The AP-1(2)-binding site had a similar combined effect (Fig. 4d).

To determine whether the AP-1 transcription factor was associated with the VEGF-D promoter in vivo, we performed ChIP assays with an AP-1-specific antibody and PCR using the primers against the regulatory elements of the VEGF-D promoter in NOZ and GBC-SD cell lines. As shown in Fig. 4 (e, f), DNA fragments covering the two AP-1 binding sites (119 bp for AP-1(1), 150 bp for AP-1(2)) were amplified by chromatin immunoprecipitation with an anti-AP-1 antibody. The same band was obtained with the input DNA, whereas the normal IgG control and no antibody control did not result in the immunoprecipitation of DNA fragments detectable by PCR amplification. Consistent with the results by EMSA, TNF-α enhanced the intensity of the anti-AP-1 band.

Taken together, these results demonstrate that the AP-1 transcription factor can bind directly to the consensus binding sites in the VEGF-D promoter region and the TNF-α can improve the combined effect.

### Upregulation of VEGF-D expression and VEGF-D promoter activity by the TNF-α/ERK1/2/AP-1 pathway

**Fig. 5:**
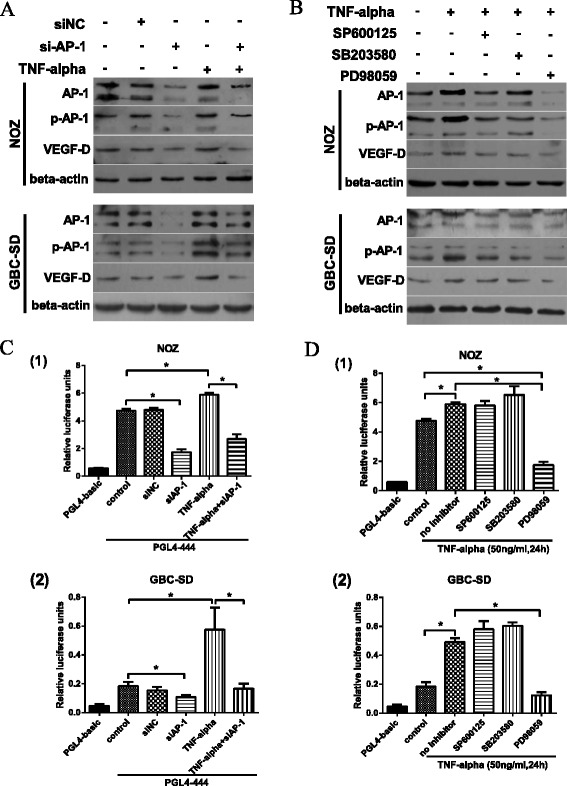
TNF-α upregulated VEGF-D expression and VEGF-D promoter activity downstream of the ERK1/2/AP-1 pathway. **a**, **c** The effect of the TNF-α⁄AP-1 signaling pathway on the promoter activity and protein expression of the VEGF-D gene. Transfection with AP-1 siRNA effectively knocked down the expression of AP-1 and p-AP-1 in both NOZ and GBC-SD cells. The protein level and promoter activity of VEGF-D were accordingly reduced irrespective of treatment with TNF-α. **b**, **d** The effect of inhibition of MAPK pathway members on the protein expression and promoter activity of VEGF-D. When treated with SP600125 (10 μM), SB203580 (20 μM) or PD98059 (50 μM), the expression of AP-1 and p-AP-1 in both NOZ and GBC-SD cells were reduced. However, the protein expression and promoter activity of VEGF-D were significantly reduced only in the PD98059-treated group. *P < 0.05

To determine the effect of the TNF-α⁄AP-1 signaling pathway on the promoter activity and protein expression of the VEGF-D gene, we measured the luciferase intensity of the PGL4-444 plasmid and VEGF-D expression in NOZ (or GBC-SD) cells treated with TNF-α or transfected with AP-1 (c-Jun) siRNA against AP-1 (siAP-1). The siAP-1 oligos effectively knocked-down the expression of AP-1 and p-AP-1 in NOZ (or GBC-SD) cells compared with the negative control and siNC groups (Fig. 5a). As shown in Fig. 5 (a, c), the protein level and promoter activity of the VEGF-D gene were significantly reduced after transfection with siAP-1. TNF-α was demonstrated to enhance the expression of AP-1, p-AP-1, and VEGF-D and to increase the luciferase activity of the VEGF-D promoter. In contrast, when NOZ (or GBC-SD) cells were transfected with siAP-1, the ability of TNF-α to upregulate the luciferase activity and the protein expression of VEGF-D were blunted.

To explore which member of the MAPK family (JNK, p38 or ERK1/2) is involved in the TNF-α ⁄AP-1/VEGF-D signaling pathway, we investigated the effects of MAPK pathway inhibitors on the protein expression of AP-1, p-AP-1, and VEGF-D and the luciferase activity of VEGF-D promoter. As shown in Fig. 5 (b, d), treatment of NOZ (or GBC-SD) cells with SP600125 (10 μM), SB203580 (20 μM) or PD98059 (50 μM) resulted in reduced expression of AP-1 and p-AP-1. However, the protein expression and promoter activity of VEGF-D were significantly reduced in the PD98059-treated group (compared with control and the TNF-α-treated groups, *P* < 0.05) but not in the SP600125- or SB203580-treated groups. Therefore, ERK1/2 is involved in the TNF-α/AP-1 signaling pathway.

Taken together, these experiments confirm the upregulation of VEGF-D expression and VEGF-D promoter activity by the TNF-α/ERK1/2/AP-1 pathway.

### The role of the TNF-α - VEGF-D axis in tube formation of HDLECs *in vitro*

**Fig. 6:**
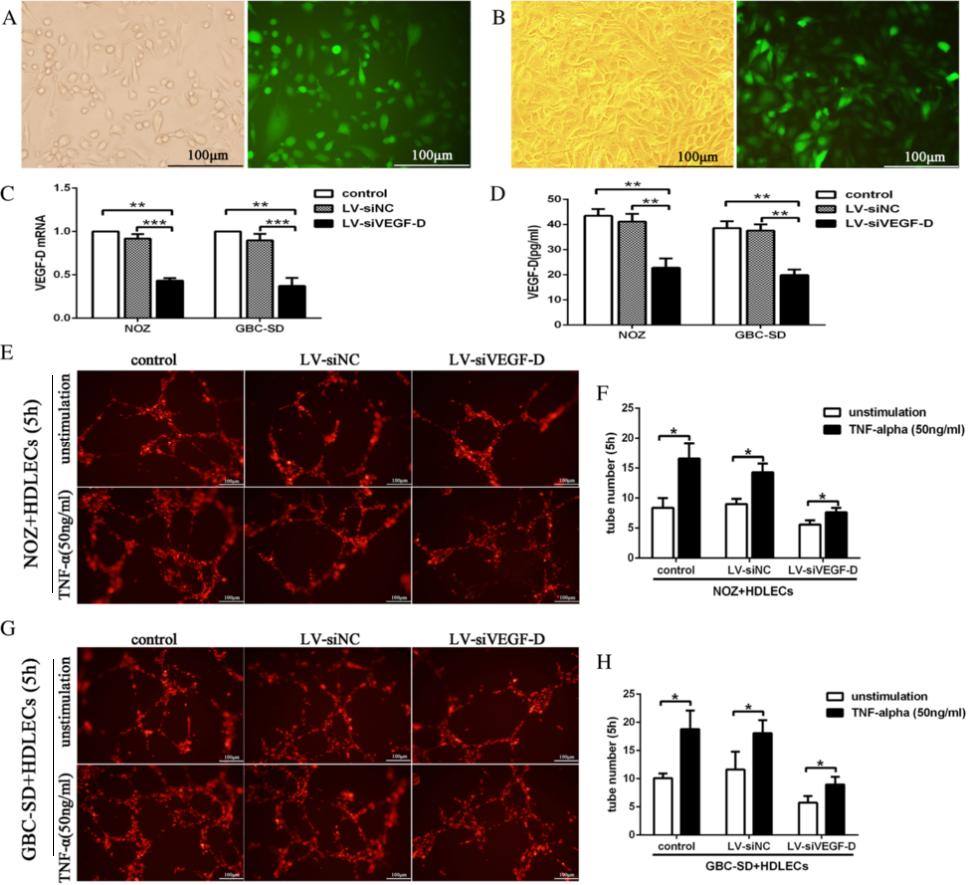
The TNF-α - VEGF-D axis promoted the tube formation of human dermal lymphatic endothelial cells (HDLECs) *in vitro*. **a**, **b** Construction of a NOZ cell line and a GBC-SD cell line stably expressing lentiviral VEGF-D shRNA and a green fluorescent protein sequence. The cells were observed under a fluorescence microscope with bright or blue light. **c**, **d** VEGF-D mRNA and protein expression of NOZ or GBC-SD cells stably transfected with LV-siVEGF-D were analyzed by real-time reverse transcription-polymerase chain reaction (RT-PCR) and enzyme-linked immunosorbent assay (ELISA), respectively. GAPDH served as an internal control. **e**, **f**, **g**, **h** DiI-labeled HDLECs (emit red fluorescence) were cocultured with the three NOZ (or GBC-SD) cell lines and were treated with TNF-α (50 ng⁄ mL) for 5 h. HDLEC tube formation was observed under fluorescence microscopy, and the tube number was counted. (*P < 0.05; **P < 0.01; ***P < 0.001)

After confirming TNF-α-induced expression of VEGF-D *in vitro*, we wanted to further analyze the role of the TNF-α-VEGF-D axis in the tube formation of HDLECs. We first established a NOZ cell line (Fig. 6a) and a GBC-SD cell line (Fig. 6b) stably expressing lentiviral VEGF-D shRNA and employed real-time PCR and ELISA to measure the efficacy of VEGF-D knockdown at the mRNA and protein level. As shown in Fig. 6 (c, d), the mRNA and protein levels of VEGF-D in the LV-siVEGF-D group (NOZ or GBC-SD cells infected with lentiviral VEGF-D shRNA) were significantly decreased (**P < 0.01, ***P < 0.001) relative to the control (NOZ or GBC-SD cells only) and LV-siNC (NOZ or GBC-SD cells infected with empty vector) groups. Subsequently, we used a three-dimensional coculture system in which the GBC cells and HDLECs were cultured together to observe the role of TNF-α and VEGF-D in the tube formation of HDLECs. HDLECs labeled by DiI were separately cocultured with three cell lines (NOZ or GBC-SD, LV-siVEGF-D and LV-siNC) on Matrigel with or without treatment with TNF-α (50 ng/ml). The phenomenon of tube formation was observed 1 h, 3 h, 5 h, 8 h, and 24 h after coculture. As shown in Fig. 6 (e, f, g, h), the greatest number of tubes was observed 5 h after cell seeding, and the tube-like structures disappeared after 24 h (data not shown). These data led us to conclude the following: (1) the tube formation of HDLECs decreased with knock-down of VEGF-D expression, and (2) the number tubes formed by HDLECs significantly increased after treatment with TNF-α, which could be impaired with knock-down of VEGF-D expression.

### The TNF-α-VEGF-D axis promotes the lymphatic metastasis of GBC *in vivo*

**Fig. 7:**
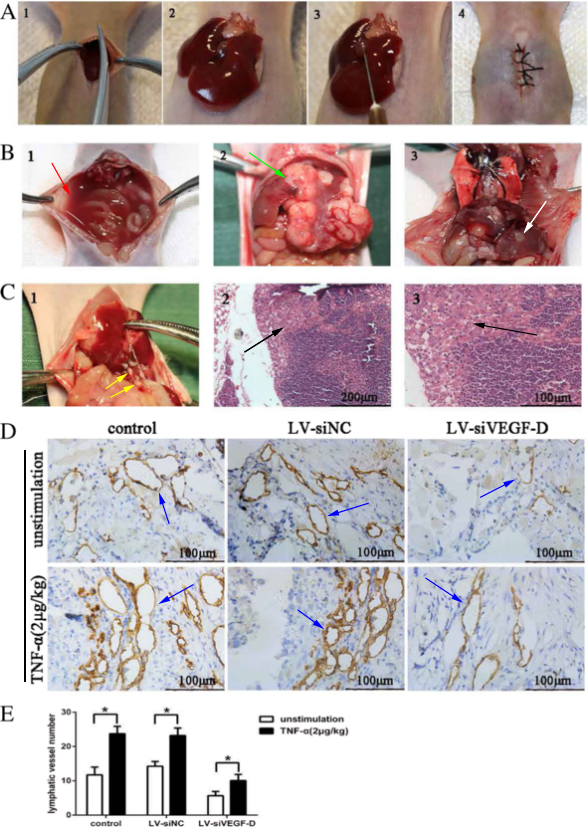
The TNF-α-VEGF-D axis is involved in lymphangiogenesis and lymph node metastasis (LNM) of GBC *in vivo*. **a**. Establishment of orthotopic xenograft models of GBC in nude mice. After anesthesia, the abdominal cavity of the nude mouse was opened, the gallbladder was exposed, and one of three NOZ cell lines (NOZ, LV-siNC, or LV-siVEGF-D) was injected into the cavity of gallbladder; the abdominal cavity was subsequently closed. **b**, **c**. After treatment with TNF-α (2 μg⁄ kg) twice a week for 3 weeks, the mice were dissected, and the tumors were excised. Infiltrative growth (green arrow), LNM (yellow arrow), ascites (red arrow) and hepatic metastasis (white arrow) were observed in the orthotopic xenograft models. LNM was further confirmed by H-E staining (C-2: 200×, C-3: 400×), and invasive tumor cells (black arrow) could be observed in the lymphoid follicles. **d**. Detection of lymphatic vessels (marked by LYVE-1 and indicated by blue arrows) in the orthotopic xenograft tumors was achieved by immunohistochemistry. **e**. Number of lymphatic vessel in the orthotopic xenograft tumors. TNF-α increased the number of lymphatic vessels in the NOZ and LV-siNC group, whereas the knockdown of VEGF-D decreased this effect (*P < 0.05)

To investigate the role of the TNF-α - VEGF-D axis in lymphangiogenesis and lymph node metastasis *in vivo*, we injected three NOZ cell lines (NOZ, LV-siNC and LV-siVEGF-D) into the gallbladders of nude mice to established three orthotopic xenograft models of GBC (Fig. 7a). Two weeks later, TNF-α (2 μg⁄kg) was injected into the abdominal cavity twice a week for 3 weeks. Lymph node metastases were observed with the naked eye and further confirmed by HE staining (Fig. 7c). The lymphatic vessels of tumors were detected by immunohistochemistry using an LYVE-1 antibody. As shown in Fig. 7b, infiltrative growth was observed in most of the orthotopic xenograft tumors. Lymph node metastases, ascites or hepatic metastases were observed in some mice, whereas lung metastases were not observed. Figure 7d demonstrates that TNF-α increased the LVD of orthotopic xenograft tumors compared with the control group, and this effect was impaired when the VEGF-D was knocked down by lentiviral-mediated shRNA (LV-siVEGF-D group). As shown in Table 2, the rates of lymph node metastasis were increased by TNF-α.

**Table 2:** Lymphatic vessel density (LVD) and lymph node metastasis (LNM) of the orthotopic xenograft tumors in nude mice

	Unstimulation		TNF-α (2 μg/kg)	
	LVD	LNM	LVD	LNM
control	11.73 ± 2.28	3/5	23.73 ± 2.17^*^	5/5
LV-siNC	14.27 ± 1.36	2/5	23.20 ± 2.18^*^	4/5
LV-siVEGF-D	5.67 ± 1.25	1/5	10.07 ± 1.83^*^	2/5

*P < 0.05

## Discussion

As previously mentioned, the relationship between inflammation and cancer was first appreciated by Vichow in 1863. It is currently estimated that approximately 25 % of the malignancies worldwide are induced by chronic inflammation [29, 30]. The characteristics of this chronic inflammation is the infiltration of a large number of inflammatory cells that secrete various cytokines [31]. TNF-α, mainly secreted by macrophage, is a key player in cancer-related inflammation. Chronic inflammation induced by gallstones, infection, or other factors is one of the leading causes of GBC according to epidemiological investigations, [32, 33] and TNF-α has been detected in the inflammatory environment of the gallbladder [34, 35]. Consistent with these reports, our laboratory recently observed that the level of TNF-α in the bile of GBC patients was higher than that of patients with cholecystic polypus (without obvious inflammation) and demonstrated the ability of TNF-α to promote lymphangiogenesis in GBC [20].

Lymphangiogenesis is thought to be an important step in cancer metastasis [36]. Our previous study have confirmed that TNF-α can promote lymphangiogenesis of GBC through upregulation of VEGF-C. Meanwhile, we found that the effect of TNF-α-induced lymphangiogenesis in GBC was only partially inhibited with knock-down of VEGF-C expression. This interesting phenomenon promoted us to speculate that there should be other molecular mechanisms involved in the TNF-α-induced lymphangiogenesis in GBC. Similar to VEGF-C, VEGF-D is another key lymphangiogenic factor which is associated with lymphangiogenesis and lymph node metastasis of GBC [21]. Therefore, we hypothesized that VEGF-D may be involved in the TNF-α-induced lymphatic metastasis of GBC.

In the present study, we found that the level of TNF-α in the bile of GBC patients was correlated with the expression of VEGF-D in the tissue. Subsequently, we confirmed *in vitro* that TNF-α significantly increased the mRNA and protein expression of VEGF-D in NOZ and GBC-SD cell lines within the dose range of 10–50 ng⁄mL in a dose- and time-dependent manner. We further to reveal that TNF-α can upregulate the protein expression and promoter activity of VEGF-D through the ERK1/2 - AP-1 pathway. Moreover, we determined that TNF-α can promote tube formation of HDLECs, lymphangiogenesis and lymph node metastasis of GBC by upregulation of VEGF-D *in vitro* and *in vivo*. In the tube formation assay, HDLECs were previously labeled by DiI before co-culture with GBC cells and observed by the inverted fluorescence microscope after co-culture. This method can effectively exclude the interference of GBC cells when observation. In addition, the orthotopic xenograft model of GBC in nude mice is more able to reflect the growth pattern of GBC in human body.

Many studies have focused on the relationship between VEGF-D and lymphatic metastasis [21, 37–40]. However, few investigations have concentrated on the regulation of VEGF-D promoter activity. To date, only two studies have suggested that orphan receptor hepatocyte nuclear factor 4α (HNF-4α), chicken ovalbumin upstream promoter transcription factors 1 and 2 (COUP-TF1 and COUP-TF2) and AP-1 bind to the VEGF-D promoter [41, 42]. A large number of studies have demonstrated that the downstream effector molecules associated with tumor progression are NF-κB or AP-1 [22, 43]. To determine whether TNF-α regulates VEGF-D promoter activity through these two transcription factors, we used the TFbind and Promoter Scan programs to search for potential binding sites of NF-κB or AP-1 in the three fragments of VEGF-D promoter with higher activities (−988 to -717 nt,-444 to -325 nt,and −154 to -57 nt), and found that the −444 to -325 nt region contains two putative AP-1 binding sites, whereas NF-κB sites were not found. Subsequently, we confirmed that both the AP-1 sites could bind to the VEGF-D promoter and that TNF-α could enhance the combination by site-directed mutagenesis, EMSA, and ChIP analysis. Further, we used siRNA to knock down AP-1, and the protein level of VEGF-D and the activity of the PGL4-444 plasmid were consequently decreased in the both groups with or without TNF-α treatment.

It is demonstrated that the multiple effects of TNF-α in cancers are due to the different downstream signaling pathways activated by the combination of TNF-α and its receptor (mainly through NF-κB and (or) AP-1 pathway). There are two AP-1 binding sites (no NF-κB site) in the core region of VEGF-D promoter, which revealed that TNF-α-induced upregulation of VEGF-D is mainly through the AP-1 pathway. Two signaling pathways associated with AP-1 have been clarified in previous studies: the TNF-α - TNFR1 - signaling complex - MAP3K (ASK1) - JNK or p38 MAPK - AP-1 pathway and the TNF-α - TNFR1 - Ras - Raf - MEK1 - ERK1/2 - AP-1 pathway [44]. To further determine which pathway is involved in the TNF-α - VEGF-D axis, we employed three reagents, SP600125, SB203580 and PD98059, to selectively inhibit JNK, p38 MAPK and ERK1/2, respectively. The protein expression of AP-1, p-AP-1, and VEGF-D and the activity of the PGL4-444 construct were significantly inhibited in the PD98059 treatment group, which indicated that TNF-α upregulated VEGF-D promoter activity and protein expression primarily through the ERK1/2/AP-1 signaling pathway.

The active Ras proteins combine with the guanosine triphosphate (GTP) and then activates the downstream signaling pathways including the MAPK pathway [45]. The alteration of Ras protein conformation caused by Ras gene mutation makes it lose the GTPase activity and leads to the continuous activation of the downstream signaling which accordingly promotes cell proliferation and invasion. K-ras gene is a member of the Ras family and K-ras mutation has been reported in various malignancies including GBC and NOZ cell line [45–48]. As mentioned above, the Ras protein is an effector between TNFR and ERK1/2. Thus we can speculate that K-ras mutation could enhance the activity of the “TNF-α/ERK1/2/AP-1/VEGF-D” pathway in NOZ cells which might accordingly enable the nude mice bearing human GBC in the present study more prone to appear lymphatic metastasis.

In this study, we first discovered the relationship between the TNF-α - VEGF-D axis and the lymphangiogenesis and lymphatic metastasis of GBC. Subsequently, we demonstrated that the regulatory mechanism between TNF-α and VEGF-D is dependent on the ERK1/2/AP-1 signaling pathway. Furthermore, we determined the core activity region of the VEGF-D promoter and identified two AP-1 binding sites in these regions. The regulatory mechanisms of inflammation-induced tumor metastasis are very complicated, but our work helps elucidate some of these mechanisms.

Together with our previous study, these results reveal that TNF-α can promote lymphangigenesis and lymph node metastasis of GBC at least by two signaling pathways: the NF-κB/VEGF-C pathway and the ERK1/2/AP-1/VEGF-D pathway. But, which pathway is dominated or both are equally important, needs further study.

## Conclusions

To our knowledge, our research represents the first report that TNF-α can promote lymphangiogenesis and lymphatic metastasis of GBC through the ERK1/2/AP-1/VEGF-D pathway.

### Availability of data and materials

The datasets supporting the conclusions of this article are included within the article.

## Abbreviations

TNF-α: Tumor necrosis factor-alpha
VEGF-D: Vascular endothelial growth factor D
GBC: Gallbladder cancer
HDLECs: Human dermal lymphatic endothelial cells
LVD: Lymphatic vessel density
LNM: Lymph node metastases
